# Workplace Bullying and Perceived Helpfulness of Psychosocial Support Among Korean Nurses Following the Anti‐Workplace Bullying Law: A Cross‐Sectional Study

**DOI:** 10.1155/jonm/1127218

**Published:** 2026-10-17

**Authors:** Eun Young Choi, Haeyoung Lee

**Affiliations:** ^1^ Red Cross College of Nursing Chung-Ang University Seoul South Korea cau.ac.kr

**Keywords:** nurses, nursing management, organizational support, workplace bullying

## Abstract

**Background:**

Workplace bullying remains an important concern in nursing management. In South Korea, workplace bullying has been prohibited under the Labor Standards Act since 2019, but evidence regarding nurses’ experiences of workplace bullying and their perceptions of support following implementation remains limited. This study examined workplace bullying among Korean nurses, perceived changes following implementation of the Anti‐Workplace Bullying Law, awareness of organizational prevention and response systems, and perceived helpfulness of psychosocial support.

**Methods:**

A cross‐sectional online survey was conducted among 306 staff nurses working in acute care or long‐term care hospitals from March to April 2022. Workplace bullying was assessed using the Korean version of the Negative Acts Questionnaire‐Revised. Data were analyzed using descriptive statistics, independent samples *t*‐tests, and chi‐square tests.

**Results:**

Overall, 25.2% of participants were classified as having been exposed to workplace bullying during the previous 6 months, with work‐related negative behaviors being the most prominent. A decrease in bullying following implementation of the law was perceived by 39.0% of nurses with recent bullying experience and 58.5% of those without recent bullying experience. Only 45.1% reported that an organizational prevention and response system was in place, while 37.9% were unsure. Annual leave or time off and peer support were rated as the most helpful support options. Nurses with recent bullying experience rated manager‐based and several in‐house support options as less helpful.

**Conclusions:**

Nurses’ perceptions of change and support following implementation of the law were not uniform according to recent bullying experience. Strengthening the visibility, clarity, and accessibility of reporting and support mechanisms, as well as providing flexible psychosocial support options may improve the practical responsiveness of workplace bullying prevention efforts.

**Implications for Nursing Management:**

Nursing managers should address work‐related negative behaviors in routine practices, such as workload allocation and information sharing, ensure that reporting and support mechanisms are accessible, and provide multiple support pathways rather than relying primarily on manager‐based support.

## 1. Introduction

Workplace bullying poses a significant challenge within the global nursing community, affecting nurses’ physical and mental health, professional well‐being, and workforce retention [1, 2]. It is defined as repeated and unwanted negative behavior directed toward individuals or groups, including work‐related bullying, personal attacks, and physical intimidation [3]. Its consequences extend to healthcare organizations through impaired teamwork and communication, reduced job satisfaction, burnout and turnover, and potential threats to patient safety and the quality of nursing care [1, 2, 4, 5]. These multifaceted consequences make workplace bullying an important nursing management concern requiring effective prevention and organizational response.

In South Korea, workplace bullying remains a serious concern among nurses [6], and studies have reported associations between workplace bullying and adverse outcomes in this population, including burnout, depressive symptoms, and turnover intention [6–8]. Beyond these adverse outcomes, workplace bullying has been discussed in relation to the hierarchical structure of nursing organizations and “Tae‐um,” a culturally specific phenomenon in Korean nursing characterized by harsh treatment and interpersonal pressure within hierarchical nurse relationships [9]. Recent Korean studies have further identified organizational culture, leadership, organizational silence, and organizational justice as factors associated with workplace bullying [10–12], highlighting the importance of organizational context in understanding workplace bullying among nurses.

Against this background, the Korean government amended the Labor Standards Act in July 2019 to introduce provisions prohibiting workplace bullying (hereafter referred to as the Anti‐Workplace Bullying Law) [13]. The amended law defines workplace bullying as “behaviors by employers or employees that cause physical or mental suffering to other employees or deteriorate the work environment beyond the appropriate scope of work by taking advantage of superiority in rank, relationship, etc. in the workplace”. Pursuant to this law, employers are obligated to investigate reported cases and take appropriate measures to protect affected employees and respond to confirmed incidents [13]. The legislation thus established a formal legal framework for preventing and responding to workplace bullying.

However, Johnson’s ecological model of workplace bullying emphasizes that prevention and response should be understood within a broader, multilevel context that extends beyond legal measures at the societal level [14]. The model conceptualizes workplace bullying as a complex and dynamic phenomenon in which antecedents and consequences operate across interconnected levels involving individuals, coworkers and managers, organizations, and the broader society [14]. From this perspective, understanding how the Anti‐Workplace Bullying Law is reflected in practice requires consideration of how its principles of prevention and response are translated into workplace practices and experiences across these interconnected levels. This is particularly relevant in nursing, where Trépanier et al.‘s integrative model applied to registered nurses highlights job characteristics, interpersonal relationships, leadership, and organizational culture as interrelated work‐environment conditions associated with workplace bullying [15].

Additionally, the ecological model emphasizes that responses to workplace bullying should address not only its antecedents but also its consequences across multiple levels [14]. In nursing, previous research has highlighted the importance of social and organizational support for nurses affected by workplace bullying [16, 17]. Accordingly, understanding which psychosocial support options nurses themselves perceive as helpful is important for assessing whether workplace responses align with their support needs.

Despite the importance of understanding how prevention, response, and support are reflected across these interconnected levels, empirical evidence remains limited. Previous studies have primarily examined the prevalence, antecedents, and consequences of workplace bullying among nurses [18, 19] or have evaluated prevention and intervention strategies [20–22].

To address these gaps, this study examined workplace bullying among nurses following implementation of the Anti‐Workplace Bullying Law, with particular attention to organizational prevention and response systems and the perceived helpfulness of psychosocial support. The study’s three main research questions are: (1) What is the prevalence of workplace bullying, and what are its characteristics among nurses? (2) How do nurses perceive changes in workplace bullying and organizational prevention and response systems following implementation of the law? (3) What psychosocial support options do nurses perceive as helpful when workplace bullying occurs, and do these perceptions differ according to recent bullying experience?

## 2. Methods

### 2.1. Study Design

This study employed a cross‐sectional design using an online survey.

### 2.2. Participants

Participants were recruited using nonprobability convenience sampling. Eligible participants were staff nurses with at least 6 months of experience working in acute care hospitals or long‐term care hospitals. An a priori sample‐size calculation was performed using G^∗^Power 3.1.9.2 software [23] for chi‐square tests. Because no prior study provided an appropriate effect‐size estimate for the planned comparisons, Cohen’s conventional medium effect size (*w* = 0.30) was used [24], with a significance level of 0.05 and a statistical power of 0.95. The calculation used 12 degrees of freedom, based on the original 13‐category classification of hospital location. Based on these parameters, the minimum required sample size was 288 participants. Allowing for potential nonresponse or incomplete responses, 306 participants were recruited and included in the final analysis.

### 2.3. Survey Questionnaire

The survey questionnaire was developed in several steps. First, the research team reviewed previous literature relevant to the study questions, including studies examining the current state of workplace bullying and response strategies [25–27] and psychosocial support strategies in healthcare institutions [28, 29]. Based on this review, the study‐specific domains and initial questionnaire items were identified and drafted. The items were then refined through iterative discussions among the research team. Subsequently, two registered nurses who were actively working in clinical practice reviewed the draft questionnaire for relevance, clarity, and comprehensibility, and the wording was revised based on their feedback. The final questionnaire consisted of four sections: exposure to workplace bullying, perceived changes following the implementation of the Anti‐Workplace Bullying Law, perceived helpfulness of psychosocial support in the event of workplace bullying, and general characteristics.

#### 2.3.1. Exposure to Workplace Bullying

Exposure to workplace bullying was measured using the Korean version of the Negative Acts Questionnaire‐Revised (NAQ‐R), originally developed by Einarsen et al. [30] and translated and validated for Korean nurses by Nam et al. [31]. The Korean version consists of 22 items across three dimensions: work‐related bullying (5 items), intimidation‐related bullying (5 items), and person‐related bullying (12 items). Participants rated the frequency of negative behaviors experienced over the past 6 months on a five‐point scale: never (1), less than once a month (2), about once a month (3), about once a week (4), and almost daily (5). For descriptive and group‐comparison analyses, overall and subscale scores were calculated as mean item scores ranging from 1 to 5, with higher scores indicating more frequent exposure to negative behaviors. Participants were classified as exposed to workplace bullying if they reported at least two negative behaviors occurring weekly or daily during the previous 6‐month period [31, 32]. All other participants were classified as not exposed. The instrument demonstrated high internal consistency, with Cronbach’s alpha of 0.93 in Nam et al. [31] and 0.94 in the present study.

#### 2.3.2. Perceived Changes Following the Implementation of the Anti‐Workplace Bullying Law

To assess perceived changes following the implementation of the Anti‐Workplace Bullying Law, measurements focused on three key domains: changes in the frequency of bullying behaviors, establishment of organizational prevention and response systems, and perceived effectiveness of preventive education in addressing workplace issues. These items were developed with reference to the literature [25–27]. Single‐item measures were used because each item was intended to capture participants’ perception of a specific aspect of the post‐implementation situation [33]. Accordingly, each item was analyzed independently, and internal consistency reliability was not assessed.

Perceived changes in the frequency of bullying behaviors since the implementation of the Anti‐Workplace Bullying Law were measured on a five‐point Likert scale (1 = greatly decreased, 2 = somewhat decreased, 3 = no change, 4 = somewhat increased, 5 = greatly increased). For analysis, responses were recategorized according to the direction of perceived change: responses 1 and 2 were recategorized as “decreased,” responses 4 and 5 as “increased,” and response 3 as “no change.”

The presence of workplace bullying prevention and response systems within healthcare institutions was measured using a three‐point categorical scale (1 = yes, 2 = no, 3 = unsure). Participants who responded “yes” were asked to identify the types of systems currently implemented in their institutions through multiple‐response questions. Specific types included: inclusion of bullying prevention and response measures in employment regulations, preventive education for staff, in‐house campaigns such as posting of prevention posters, CEO declarations to eradicate workplace bullying, reporting and investigation procedures within the institution, disciplinary or personnel action standards for perpetrators, and operation of counseling programs for affected workers.

Additionally, the perceived effectiveness of workplace bullying prevention education for healthcare institution staff was measured on a four‐point scale (1 = very helpful, 2 = helpful, 3 = somewhat helpful, 4 = not helpful at all). For analysis, responses 1 through 3, all of which indicated some degree of perceived helpfulness, were recategorized as “helpful,” and response 4 as “not helpful.”

#### 2.3.3. Perceived Helpfulness of Psychosocial Support When Exposed to Workplace Bullying

Nine types of psychosocial support options were developed with reference to support resources and strategies reported in previous studies [28, 29]. Participants rated the perceived helpfulness of each support type in the event of workplace bullying. Responses were measured on a four‐point Likert scale (1 = not useful, 2 = somewhat useful, 3 = useful, 4 = very useful). The nine options represented distinct forms of support and were analyzed individually. No total score was calculated, and internal consistency reliability was therefore not assessed.

#### 2.3.4. General Characteristics

General characteristics collected included gender, age, educational level, hospital location, hospital type, hospital size, work unit, shift work status, and years of nursing experience. For analysis, the original location categories were collapsed into capital area (Seoul, Incheon, and Gyeonggi) and noncapital area because several regional categories contained small numbers of participants.

### 2.4. Data Collection

Data were collected through an anonymous online survey conducted by a professional survey agency from March to April 2022. A recruitment announcement was posted on online communities and social network services frequently used by nurses, including an explanation of the study purpose and content, along with a link to the survey. Because recruitment was conducted through open online postings, the number of individuals who viewed the recruitment announcement was not available; therefore, a response rate could not be calculated. To prevent duplicate participation, the survey was designed to restrict multiple responses from the same IP address. The survey took approximately 20 min to complete, and participants were provided with a gift card worth 10,000 Korean won (approximately $8 USD) upon completion.

### 2.5. Data Analysis

Data were analyzed using the IBM SPSS Statistics Version 30 (IBM Corp., Armonk, NY, USA). Participants’ general characteristics and NAQ‐R scores were analyzed using descriptive statistics. Differences in general characteristics, NAQ‐R scores, perceived changes since implementation of the Anti‐Workplace Bullying Law, and perceived helpfulness of psychosocial support according to exposure to workplace bullying were analyzed using independent *t*‐tests and chi‐square tests. Effect sizes were reported as Cramér’s V for chi‐square tests and Cohen’s d for independent *t*‐tests. No adjustment for multiple comparisons was applied because each comparison addressed a distinct outcome or characteristic and was interpreted independently. The psychosocial support options were also treated as distinct item‐level outcomes rather than indicators of a single underlying construct.

### 2.6. Ethical Considerations

This study was approved by the Institutional Review Board at the author’s affiliation (IRB No. 1041078–202109‐HR‐297‐01C). Informed consent was obtained from the participants after they were provided with an explanation of the study background and purpose, methods, anticipated risks and inconveniences associated with study participation, benefits of study participation, confidentiality, voluntary participation and the right to withdraw, and the rights of the study participants at the initial screening.

## 3. Results

### 3.1. Participant Characteristics

Table 1 presents the general characteristics of the participants. A total of 306 staff nurses completed the survey. The majority (*n* = 294, 96.1%) were female, with a mean age of 32.76 years (SD = 5.32). A total of 223 (72.9%) had a bachelor’s degree. Most participants worked in hospitals located in the capital area (*n* = 237, 77.5%). The most common hospital type was an advanced general hospital (*n* = 113, 36.9%), and the least common was a long‐term care hospital (*n* = 27, 8.8%). The most common hospital size was 500 beds or more (*n* = 152, 49.7%), and the most common work unit was the general ward (*n* = 164, 53.6%). Most participants worked shifts (*n* = 196, 64.1%), with a mean number of years of nursing experience of 6.74 years (SD = 4.18).

**TABLE 1:** Participant characteristics.

Characteristics		Total (*n* = 306)		Group without bullying (*n* = 229)		Group with bullying (*n* = 77)		*χ* ^2^ (*p*)	Effect size (Cramér’s V)
		*n*	%	*n*	%	*n*	%		
Gender	Male	12	3.9	9	3.9	3	3.9	0.000 (0.989)	< 0.01
	Female	294	96.1	220	96.1	74	96.1		

Age	20–29 years	104	34.0	75	32.8	29	37.7	3.148 (0.207)	0.10
	30–39 years	160	52.3	118	51.5	42	54.5		
	≥ 40 years	42	13.7	36	15.7	6	7.8		

Education level	Associate	53	17.3	45	19.7	8	10.4	4.407 (0.110)	0.12
	Bachelor	223	72.9	160	69.9	63	81.8		
	Graduate	30	9.8	24	10.5	6	7.8		

Hospital location	Capital area^†^	237	77.5	177	77.3	60	77.9	0.013 (0.909)	< 0.01
	Non‐capital area	69	22.5	52	22.7	17	22.1		

Hospital type	Advanced general hospital	113	36.9	86	37.6	27	35.1	3.533 (0.317)	0.11
	General hospital	99	32.4	72	31.4	27	35.1		
	Hospital	67	21.9	54	23.6	13	16.9		
	Long‐term care hospital	27	8.8	17	7.4	10	13.0		

Hospital size	< 100 beds	50	16.3	40	17.5	10	13.0	0.962 (0.915)	0.06
	100‐299 beds	72	23.5	53	23.1	19	24.7		
	300‐499 beds	32	10.5	23	10.0	9	11.7		
	500‐999 beds	84	27.5	62	27.1	22	28.6		
	≥ 1000 beds	68	22.2	51	22.3	17	22.1		

Ward	General ward	164	53.6	116	50.7	48	62.3	3.229 (0.199)	0.10
	Outpatient unit	62	20.3	50	21.8	12	15.6		
	ICU/ER	80	26.1	63	27.5	17	22.1		

Work type	Shift work	196	64.1	139	60.7	57	74.0	4.445 (0.035)	0.12
	No shift work	110	35.9	90	39.3	20	26.0		

Years of nursing experience	< 2 years	33	10.8	17	7.4	16	20.8	13.991 (0.003)	0.21
	2–5 years	80	26.1	59	25.8	21	27.3		
	5–10 years	118	38.6	89	38.9	29	37.7		
	≥ 10 years	75	24.5	64	27.9	11	14.3		

Abbreviations: ER, emergency room; ICU, intensive care unit.

^†^Capital area included Seoul, Incheon, and Gyeonggi.

Exposure to workplace bullying in the past 6 months differed significantly according to work type (*χ* ^2^ = 4.445, *p* = 0.035) and years of nursing experience (*χ* ^2^ = 13.991, *p* = 0.003). The proportion of nurses working shifts (74%) and the percentage of nurses with less than 2 years of nursing experience (20.8%) were higher in the group exposed to workplace bullying.

### 3.2. Exposure to Workplace Bullying

The overall NAQ‐R mean item score ranged from 1.00 to 4.14 (*M* = 1.68, SD = 0.62). Across the NAQ‐R subscales, work‐related bullying showed the highest mean item score (*M* = 2.05, SD = 0.74), followed by person‐related bullying (*M* = 1.68, SD = 0.73) and intimidation‐related bullying (*M* = 1.33, SD = 0.51). Participants classified as exposed to workplace bullying showed significantly higher overall and subscale mean item scores than those not exposed (Table 2).

**TABLE 2:** NAQ‐R mean item scores by workplace bullying exposure status.

Dimensions	Total (*n* = 306)	Group without bullying (*n* = 229)	Group with bullying (*n* = 77)	*t* (*p*)	Effect size (Cohen’s d)
	Mean ± SD	Mean ± SD	Mean ± SD		
Overall	1.68 ± 0.62	1.42 ± 0.34	2.46 ± 0.61	−14.24 (< 0.001)	2.44
Work‐related	2.05 ± 0.74	1.77 ± 0.54	2.87 ± 0.64	−14.71 (< 0.001)	1.94
Intimidation‐related	1.33 ± 0.51	1.17 ± 0.29	1.81 ± 0.68	−11.65 (< 0.001)	1.53
Person‐related	1.68 ± 0.73	1.38 ± 0.40	2.57 ± 0.77	−17.36 (< 0.001)	2.29

*Note:* Total and subscale scores are presented as mean item scores, with possible scores ranging from 1 to 5. Higher scores indicate more frequent exposure to negative behaviors.

Abbreviations: NAQ‐R, Negative Acts Questionnaire–Revised; SD, standard deviation.

At the item level, exposure was examined in terms of both the proportion of participants reporting each behavior at least once during the preceding 6 months (i.e., any response other than “never”) and the mean frequency rating on the five‐point scale. The five most frequently reported negative behaviors were “Being exposed to an unmanageable workload” (73.9%, *M* = 2.67, SD = 1.32), “Someone withholding information which affects your performance” (69.0%, *M* = 2.27, SD = 1.03), “Having key areas of responsibility removed or replaced with more trivial or unpleasant tasks” (57.2%, *M* = 1.92, SD = 0.99), “Being shouted at or being the target of spontaneous anger” (55.2%, *M* = 1.93, SD = 1.05), and “Having your opinions ignored” (51.6%, *M* = 1.75, SD = 0.89). “Being exposed to an unmanageable workload” also showed the highest mean frequency rating among all 22 items, with 29.4% of participants experiencing it on a weekly or daily basis, whereas the remaining four items were experienced on a weekly or daily basis by 4.9%–12.4% of participants (Supporting Table S1).

### 3.3. Perceived Changes Following the Implementation of the Anti‐Workplace Bullying Law

Table 3 presents participants’ perceived changes following the implementation of the Anti‐Workplace Bullying Law. Perceptions of changes in the frequency of bullying behaviors differed markedly between groups. Among participants exposed to workplace bullying, 61.0% reported no change in bullying behaviors, whereas 58.5% of those without recent workplace bullying exposure reported a decrease in such behaviors (*χ* ^2^ = 8.859, *p* = 0.003).

**TABLE 3:** Perceived changes following the implementation of the Anti‐Workplace Bullying Law.

Variables	Total (*n* = 306)		Group without bullying (*n* = 229)		Group with bullying (*n* = 77)		*χ* ^2^ (*p*)	Effect size (Cramér’s V)
	*n*	(%)	*n*	(%)	*n*	(%)		
*Changes in the frequency of bullying behaviors* ^†^								
Decreased	164	(53.6)	134	(58.5)	30	(39.0)	8.859 (0.003)	0.17
No change	142	(46.4)	95	(41.5)	47	(61.0)		

*Establishment of organizational prevention and response systems*								
Yes	138	(45.1)	109	(47.6)	29	(37.7)	2.808 (0.246)	0.10
No	52	(17.0)	39	(17.0)	13	(16.9)		
Unsure	116	(37.9)	81	(35.4)	35	(45.5)		

*Components of the prevention and response system (multiple responses)*								
Preventive education for staff	117	(38.2)	92	(40.2)	25	(32.5)	1.449 (0.229)	0.07
In‐house campaigns (e.g., prevention posters)	80	(26.1)	64	(27.9)	16	(20.8)	1.534 (0.216)	0.07
Reporting and investigation procedures	77	(25.2)	62	(27.1)	15	(19.5)	1.765 (0.184)	0.08
Inclusion in employment regulations	59	(19.3)	48	(21.0)	11	(14.3)	1.650 (0.199)	0.07
Disciplinary or personnel action standards for perpetrators	53	(17.3)	43	(18.8)	10	(13.0)	1.349 (0.245)	0.07
Counseling programs for affected workers	40	(13.1)	33	(14.4)	7	(9.1)	1.435 (0.231)	0.07
CEO declaration to eradicate workplace bullying	17	(5.6)	12	(5.2)	5	(6.5)	0.173 (0.678)	0.02

*Effectiveness of bullying prevention education*								
Helpful	169	(55.2)	137	(59.8)	32	(41.6)	7.776 (0.005)	0.16
Not helpful	137	(44.8)	92	(40.2)	45	(58.4)		

^†^No participants selected the “increased” category.

Regarding the establishment of organizational prevention and response systems, 138 participants (45.1%) reported that such systems were in place. Among the components identified, preventive education for staff was reported most frequently (38.2%), followed by in‐house campaigns such as posting prevention posters (26.1%) and reporting and investigation procedures within the institution (25.2%). Counseling programs for affected workers (13.1%) and CEO declarations to eradicate workplace bullying (5.6%) were reported least frequently. No statistically significant differences were found between groups for any component.

Perceptions of the effectiveness of preventive education also differed between groups. Among participants exposed to workplace bullying, only 41.6% reported that preventive education was helpful in addressing the problem, compared to 59.8% of those not exposed (*χ* ^2^ = 7.776, *p* = 0.005).

### 3.4. Perceived Helpfulness of Psychosocial Support When Exposed to Workplace Bullying

Table 4 presents participants’ ratings of the perceived helpfulness of psychosocial support when exposed to workplace bullying. The support type rated as most helpful by the largest proportion of participants was “availability of annual leave or time off.” Responses to other support types differed between groups. Among participants without recent workplace bullying exposure, more than half reported that eight of the nine support types would be helpful. Among those exposed to workplace bullying, higher proportions endorsed access to peer nurses for open discussion and emotional support, 24‐h accessible confidential counseling programs, and free external counseling programs outside the hospital. The support type showing the greatest difference between groups was access to nurse managers or supervisors to discuss the situation, with only 24.7% of the exposed group reporting that this support would be helpful (*χ* ^2^ = 12.001, *p* = 0.001). Additionally, the exposed group showed significantly lower proportions reporting that the following support options would be helpful than the non‐exposed group: educational programs on coping strategies for similar situations (*χ* ^2^ = 4.447, *p* = 0.035), provision of a designated space or area for self‐calming and recovery (*χ* ^2^ = 5.070, *p* = 0.024), and in‐house counseling programs with professional counselors within the hospital (*χ* ^2^ = 3.905, *p* = 0.048).

**TABLE 4:** Perceived helpfulness of psychosocial support when exposed to workplace bullying.

Support type	Total (*n* = 306)	Group without bullying (*n* = 229)	Group with bullying (*n* = 77)	*χ* ^2^ (*p*)	Effect size (Cramér’s V)
	Useful or very useful *n* (%)	Useful or very useful *n* (%)	Useful or very useful *n* (%)		
1. Provision of a designated space or area for self‐calming and recovery	165 (53.9)	132 (57.6)	33 (42.9)	5.070 (0.024)	0.13
2. Access to peer nurses for open discussion and emotional support	237 (77.5)	175 (76.4)	62 (80.5)	0.555 (0.456)	0.04
3. Access to nurse managers or supervisors to discuss the situation	127 (41.5)	108 (47.2)	19 (24.7)	12.001 (0.001)	0.20
4. In‐house counseling programs with professional counselors within the hospital	153 (50.0)	122 (53.3)	31 (40.3)	3.905 (0.048)	0.11
5. Free external counseling programs outside the hospital	164 (53.6)	121 (52.8)	43 (55.8)	0.209 (0.647)	0.03
6.24‐h accessible confidential counseling program	184 (60.1)	133 (58.1)	51 (66.2)	1.599 (0.206)	0.07
7. Availability of annual leave or time off	262 (85.6)	197 (86.0)	65 (84.4)	0.121 (0.728)	0.02
8. Stress management programs or training (e.g., mindfulness, resilience building)	171 (55.9)	131 (57.2)	40 (51.9)	0.646 (0.422)	0.05
9. Educational programs on coping strategies for similar situations	155 (50.7)	124 (54.1)	31 (40.3)	4.447 (0.035)	0.12

## 4. Discussion

This study examined workplace bullying among Korean nurses and their perceptions of organizational responses and psychosocial support following the implementation of the Anti‐Workplace Bullying Law. Approximately one‐quarter of participants reported recent workplace bullying, with work‐related negative behaviors being most prominent. Nurses with recent bullying experience were less likely to perceive a decrease in bullying, and fewer than half of participants reported that organizational prevention and response systems were in place. Among the psychosocial support options, time off and peer support were rated highly, whereas discussing the situation with a nurse manager or supervisor was rated least helpful among nurses with recent bullying experience. These findings provide a nurse‐centered perspective on workplace bullying and organizational support following the implementation of the Anti‐Workplace Bullying Law. They are consistent with Johnson’s ecological model, which views workplace bullying and responses to it across interconnected levels, and also align with the integrative model described by Trépanier et al. [14, 15].

Approximately one‐quarter of participants were classified as exposed to workplace bullying, with unmanageable workload and withholding of task‐relevant information most frequently reported. Similar work‐related negative behaviors have been reported among nurses in Australia [34]. Workload distribution has been described as an area of conflict and negotiation involving competing priorities, status, authority, and power [34], while hierarchical organizational culture has been associated with workplace bullying among Korean nurses [35]. Bullying may therefore be expressed through routine work processes, such as workload assignment and information sharing, making the boundary between legitimate work demands and workplace bullying difficult to establish [36]. This creates a particular challenge for implementation of the Anti‐Workplace Bullying Law, which requires determining whether conduct exceeds the appropriate scope of work [13]. This ambiguity may be particularly relevant to “tae‐um,” in which harsh treatment may be framed as instruction within senior–junior relationships [9]. Accordingly, case‐based organizational guidance that applies statutory criteria to common nursing situations and links them to reporting and response procedures may facilitate more consistent recognition and management of work‐related bullying.

Nurses with recent bullying experience were less likely than those without such experience to perceive a decrease in bullying following implementation of the law. This group difference may partly reflect differences in how workplace bullying is recognized. Work‐related behaviors such as excessive workload or withholding of task‐relevant information can be difficult to distinguish from routine work demands [36], and nurses without recent bullying experience may therefore have been less likely to recognize such behaviors as workplace bullying. Differences in clinical experience may also have shaped perceptions of change. Nurses in their first 2 years were highly represented in the exposed group and would have entered practice after enactment, giving them a different reference point from nurses who had experienced both pre‐ and post‐legislation work environments. However, because perceived change was assessed using a single self‐report item, the findings should be interpreted as participants’ perceptions rather than evidence of actual changes in the prevalence or severity of workplace bullying.

More than half of the participants reported either that no prevention and response system existed or that they did not know whether one did. Among the components identified, inclusion in employment regulations and reporting and investigation procedures were recognized less often than preventive education or in‐house campaigns, although both are obligations imposed on employers by the law [13]. The present data cannot determine which of these factors accounts for the low recognition, but both warrant attention. Where the provisions have not been established, compliance with the statutory requirements needs to be verified at the institutional level. Where they have been established but remain unrecognized, the system cannot be used as intended [36]. Prevention and response systems accordingly require sustained communication rather than one‐time notification, and staff recognition of them warrants review alongside their establishment.

Although preventive education was the component most often reported as being in place, nurses with recent bullying experience rated it as helpful less often than those without such experience. Anti‐workplace bullying interventions in nursing have tended to be delivered uniformly regardless of individual nurses’ experiences, and have concentrated on prevention rather than supporting nurses who have already been affected [20]. Education framed primarily for nurses who have not encountered bullying may have been of somewhat less value for those who had. Content may be a further consideration, since education drawing on situations more severe than those nurses typically encounter may provide limited guidance for recognizing ambiguous behaviors as bullying [36]. To be useful for nurses who have already experienced bullying, preventive education may need to address the support available after an incident as well as prevention, and to incorporate interactive approaches that allow nurses to practice responses to situations encountered in clinical practice [21, 22]. Future research should examine the content and delivery of workplace bullying prevention education in healthcare institutions and evaluate its effects according to nurses’ bullying experience.

Among the psychosocial support options, annual leave or time off was rated as helpful most often, followed by emotional support from peer nurses, with neither differing by bullying experience. Time away from work may facilitate recovery by allowing psychological detachment from work and greater control over nonwork time [37]. Although the Labor Standards Act requires employers to provide paid leave or other protective measures during an investigation and following confirmation of workplace bullying [13], access to such measures is tied to formal recognition of an incident. The preference for time off in this study suggests that recovery needs may arise before, or independently of, formal organizational procedures. Flexible leave or temporary scheduling adjustments may therefore be useful as supportive options that do not depend on completion of a bullying investigation. Emotional support from peer nurses was also rated highly. Social support has been identified as an important coping mechanism among nurses experiencing workplace bullying [16], and peer‐support mechanisms have been proposed as part of organizational responses to bullying [17]. Peer‐based interventions have also been implemented in nursing to prepare nurses to support colleagues and intervene when bullying occurs [38]. Incorporating trained peer supporters into organizational response systems may therefore make this form of support more consistently available to nurses experiencing bullying.

Discussing the situation with a nurse manager or supervisor was rated as helpful least often among nurses with recent bullying experience and showed the largest group difference among the psychosocial support options. Nurse managers may occupy a dual role in workplace bullying situations, providing support to affected staff while also receiving reports and facilitating organizational processes for assessment, resolution, and follow‐up [39]. This combination of supportive and procedural responsibilities may affect how manager‐based support is perceived, particularly when confidentiality or impartiality is a concern [36]. In addition, manager‐based support may be inappropriate or unavailable when managers or other nurses in supervisory positions are themselves involved in bullying [16, 34]. These considerations suggest that manager preparation may need to emphasize impartial responses, confidentiality, and supportive communication, while organizations should provide alternative reporting and support pathways when the immediate manager is involved in, or too closely connected to, the situation.

This study has several limitations. First, bullying experiences were assessed retrospectively over the previous 6 months, which may have introduced recall bias. Second, voluntary recruitment through an online platform may have resulted in selection bias and limited the representativeness and generalizability of the sample. Third, the cross‐sectional design precludes causal inference and does not allow conclusions regarding the effectiveness of the Anti‐Workplace Bullying Law. In addition, the study‐specific single‐item measures of perceived change require further validation; accordingly, these findings should be interpreted as participants’ perceptions rather than evidence of actual changes following implementation of the law. Fourth, organizational prevention and response systems were assessed through nurses’ awareness and perceptions rather than direct organizational audits or records, and therefore the findings cannot establish whether such systems were objectively present or adequately implemented. Finally, the descriptive and bivariate analyses did not examine factors independently associated with workplace bullying or support perceptions. Future studies using longitudinal or repeated assessments, organizational‐level data, and multivariable analyses in more diverse samples are warranted.

## 5. Conclusions

This study found that workplace bullying was reported by approximately one‐quarter of Korean nurses, with work‐related negative behaviors being the most prevalent form. Nurses’ perceptions of changes following implementation of the Anti‐Workplace Bullying Law and the helpfulness of preventive education and psychosocial support varied according to recent bullying experience, indicating that these perceptions were not shared uniformly across nurses. The results highlight the importance of strengthening the visibility, clarity, and accessibility of reporting and support mechanisms, as well as providing multiple and flexible psychosocial support options to improve the practical responsiveness of workplace bullying prevention efforts in nursing practice.

## 6. Implications for Nursing Management

Nursing managers should ensure that workplace bullying prevention, reporting, and support mechanisms are clearly communicated and readily accessible to staff. Given the prominence of work‐related negative behaviors, attention should also be given to routine managerial practices such as workload allocation and information sharing. Because nurses with recent bullying experience perceived manager‐based support as less helpful, organizations should provide multiple support options rather than relying on managers as the primary source of support. Nursing management should therefore establish multiple and flexible support pathways, including peer support, flexible leave, and confidential or external counseling, so that nurses experiencing bullying can access support that is appropriate to their needs.

## Funding

This work was supported by the Basic Science Research Program through the National Research Foundation of Korea (NRF), funded by the Ministry of Education (grant number 2021R1F1A1046718).

## Conflicts of Interest

The authors declare no conflicts of interest.

## Supporting Information

Additional supporting information can be found online in the Supporting Information section.

## Supporting information


**Supporting Information** Supporting Table S1. Item‐level distribution and mean frequency scores of the Negative Acts Questionnaire – Revised (NAQ‐R) (*N* = 306).

## Data Availability

The data that support the findings of this study are available from the corresponding author upon reasonable request. The data are not publicly available due to privacy and ethical restrictions.
