# Supplementary material for: Workplace Bullying and Perceived Helpfulness of Psychosocial Support Among Korean Nurses Following the Anti‐Workplace Bullying Law: A Cross‐Sectional Study
Source: J Nurs Manag. 2026 Oct 17;2026:1127218. doi: 10.1155/jonm/1127218 (PMC13675909; doi:10.1155/jonm/1127218)
Supplement: Supplementary file 1 [file JONM-2026-1127218-s001.docx]

**Supplementary Table S1. Item-level distribution and mean frequency scores of the Negative Acts Questionnaire – Revised (NAQ-R) (N=306)**

| Dimensions | Items | Mean ± SD | Response distribution | | | | |
| --- | --- | --- | --- | --- | --- | --- | --- |
|  |  |  | Never | Now and then | Monthly | Weekly | Daily |
| Work-related  bullying | 1. Someone withholding information which affects your performance | 2.27 ± 1.03 | 31.0% | 34.0% | 22.5% | 11.4% | 1.0% |
|  | 3. Being ordered to do work below your level of competence | 1.77 ± 1.02 | 54.6% | 22.2% | 16.3% | 4.9% | 2.0% |
|  | 4. Having key areas of responsibility removed or replaced with more trivial or unpleasant tasks | 1.92 ± 0.99 | 42.8% | 30.7% | 18.6% | 6.9% | 1.0% |
|  | 16. Being given tasks with unreasonable deadlines | 1.69 ± 0.94 | 55.9% | 25.5% | 13.4% | 3.9% | 1.3% |
|  | 21. Being exposed to an unmanageable workload | 2.67 ± 1.32 | 26.1% | 20.6% | 23.9% | 19.3% | 10.1% |
| Intimidation-related  bullying | 9. Intimidating behaviors such as finger-pointing, invasion of personal space, shoving, blocking your way | 1.33 ± 0.74 | 79.1% | 12.4% | 5.6% | 2.3% | 0.7% |
|  | 10. Hints or signals from others that you should quit your job | 1.23 ± 0.61 | 84.0% | 10.8% | 3.6% | 1.3% | 0.3% |
|  | 15. Practical jokes carried out by people you don’t get along with | 1.26 ± 0.65 | 82.0% | 12.4% | 3.3% | 2.0% | 0.3% |
|  | 19. Pressure not to claim something to which by right you are entitled (e.g. sick leave, holiday entitlement, travel expenses) | 1.55 ± 0.90 | 64.1% | 22.9% | 9.2% | 1.6% | 2.3% |
|  | 22. Threats of violence or physical abuse or actual abuse | 1.26 ± 0.66 | 83.0% | 9.8% | 5.9% | 0.7% | 0.7% |
| Person-related  bullying | 2. Being humiliated or ridiculed in connection with your work | 1.72 ± 0.94 | 53.3% | 29.1% | 10.8% | 5.9% | 1.0% |
|  | 5. Spreading of gossip and rumours about you | 1.64 ± 0.87 | 56.5% | 27.8% | 11.1% | 4.2% | 0.3% |
|  | 6. Being ignored or excluded | 1.66 ± 0.97 | 58.5% | 25.5% | 8.8% | 5.6% | 1.6% |
|  | 7. Having insulting or offensive remarks made about your person, attitudes or your private life | 1.60 ± 0.93 | 61.8% | 24.2% | 8.8% | 2.9% | 2.3% |
|  | 8. Being shouted at or being the target of spontaneous anger | 1.93 ± 1.05 | 44.8% | 29.7% | 15.7% | 7.5% | 2.3% |
|  | 11. Repeated reminders of your errors or mistakes | 1.78 ± 0.96 | 51.3% | 26.5% | 15.7% | 5.9% | 0.7% |
|  | 12. Being ignored or facing a hostile reaction when you approach | 1.74 ± 1.03 | 57.2% | 22.2% | 11.4% | 7.8% | 1.3% |
|  | 13. Persistent criticism of your errors or mistakes | 1.57 ± 0.87 | 63.4% | 20.9% | 11.4% | 3.9% | 0.3% |
|  | 14. Having your opinions ignored | 1.75 ± 0.89 | 48.4% | 34.6% | 12.1% | 3.9% | 1.0% |
|  | 17. Having allegations made against you | 1.58 ± 0.84 | 60.8% | 24.2% | 12.1% | 2.3% | 0.7% |
|  | 18. Excessive monitoring of your work | 1.65 ± 0.93 | 59.2% | 23.2% | 11.8% | 5.2% | 0.7% |
|  | 20. Being the subject of excessive teasing and sarcasm | 1.54 ± 0.90 | 66.0% | 19.9% | 9.2% | 3.6% | 1.3% |
| Note: Response options: never (1), now and then (2), monthly (3), weekly (4), daily (5). | | | | | | | |
